# The role of vascular smooth muscle cells in the development of aortic aneurysms and dissections

**DOI:** 10.1111/eci.13697

**Published:** 2021-11-21

**Authors:** Karlijn B. Rombouts, Tara A. R. van Merrienboer, Johannes C. F. Ket, Natalija Bogunovic, Jolanda van der Velden, Kak Khee Yeung

**Affiliations:** ^1^ Department of Surgery Amsterdam University Medical Centers Amsterdam Cardiovascular Sciences Location VU Medical Center and AMC Amsterdam The Netherlands; ^2^ Department of Physiology Amsterdam University Medical Centers Amsterdam Cardiovascular Sciences Location VU Medical Center Amsterdam The Netherlands; ^3^ Medical Library Vrije Universiteit Amsterdam The Netherlands; ^4^ Laboratory of Experimental Cardiology, Department of Cardiology Leiden University Medical Center Leiden The Netherlands

**Keywords:** aortic aneurysm, aortic dissection, pathophysiology, vascular biology, vascular smooth muscle cell

## Abstract

**Background:**

Aortic aneurysms (AA) are pathological dilations of the aorta, associated with an overall mortality rate up to 90% in case of rupture. In addition to dilation, the aortic layers can separate by a tear within the layers, defined as aortic dissections (AD). Vascular smooth muscle cells (vSMC) are the predominant cell type within the aortic wall and dysregulation of vSMC functions contributes to AA and AD development and progression. However, since the exact underlying mechanism is poorly understood, finding potential therapeutic targets for AA and AD is challenging and surgery remains the only treatment option.

**Methods:**

In this review, we summarize current knowledge about vSMC functions within the aortic wall and give an overview of how vSMC functions are altered in AA and AD pathogenesis, organized per anatomical location (abdominal or thoracic aorta).

**Results:**

Important functions of vSMC in healthy or diseased conditions are apoptosis, phenotypic switch, extracellular matrix regeneration and degradation, proliferation and contractility. Stressors within the aortic wall, including inflammatory cell infiltration and (epi)genetic changes, modulate vSMC functions and cause disturbance of processes within vSMC, such as changes in TGF‐β signalling and regulatory RNA expression.

**Conclusion:**

This review underscores a central role of vSMC dysfunction in abdominal and thoracic AA and AD development and progression. Further research focused on vSMC dysfunction in the aortic wall is necessary to find potential targets for noninvasive AA and AD treatment options.

## 1 INTRODUCTION

Aortic aneurysms (AA) are defined as a progressive weakening of the aortic wall, leading to gradual dilatation. Based on their anatomical location, AA are classified into thoracic aorta aneurysms (TAA) and abdominal aortic aneurysms (AAA), or thoraco‐abdominal AA (Crawford classification) when affecting both locations. Both TAA and AAA share some common pathophysiological characteristics and risk factors, including ageing, male sex and smoking.^1^, ^2^ Moreover, TAA is caused by inherited gene mutations in approximately 20% of the patients.^3^ In addition to the dilation of the aorta, patients may present with aortic dissections (AD). AD are defined as a tear within the intimal layer of the aorta, causing separation of the intimal and medial layers of the aortic wall. AD are divided into Stanford type A (involving the aortic arch) and type B (aortic disease starts after the left subclavian artery). The aorta of AD patients can likely dilate and develop into AA.^4^

The majority of AA patients is diagnosed accidently or after sudden rupture of the aortic wall. Sudden AA rupture is associated with an overall high mortality of 90% due to internal bleeding complications.^5^ AD are usually diagnosed acutely; commonly patients report an acute pain between the shoulders. Currently, surgical open or endovascular repair is offered to AA and AD patients with known risk of complications or rupture, mainly based on aneurysm size.^6^ Patients are usually also treated with medications for overall cardiovascular risk management. However, the majority of patients with acute type B AD is usually non‐responsive to pharmacological therapy.^7^ No further pharmaceutical treatment for prevention, stabilization or reversal of AA and AD is available, since the knowledge about the pathophysiology of aortic wall weakening is limited.

Aortic wall weakening is characterized by loss of vascular smooth muscle cells (vSMCs) due to apoptosis and extracellular matrix (ECM) degradation within the middle layer of the aortic wall. Vascular SMCs are the predominant cell type in the middle aortic layer, called the tunica media. Their embryonic origin is dependent on the location in the aortic wall; in the ascending aorta and the aortic arch, vSMCs are neural crest‐derived, whereas in the descending aortic vSMC originate from somatic mesoderm.^8^ vSMCs are paramount for providing structural and functional integrity of the aortic wall and ECM synthesis. They can adapt to environmental stimuli and mechanical stress due to their characteristic vSMC plasticity ‐ being able to switch between a contractile and a synthetic phenotype.^9^

**FIGURE 1:**
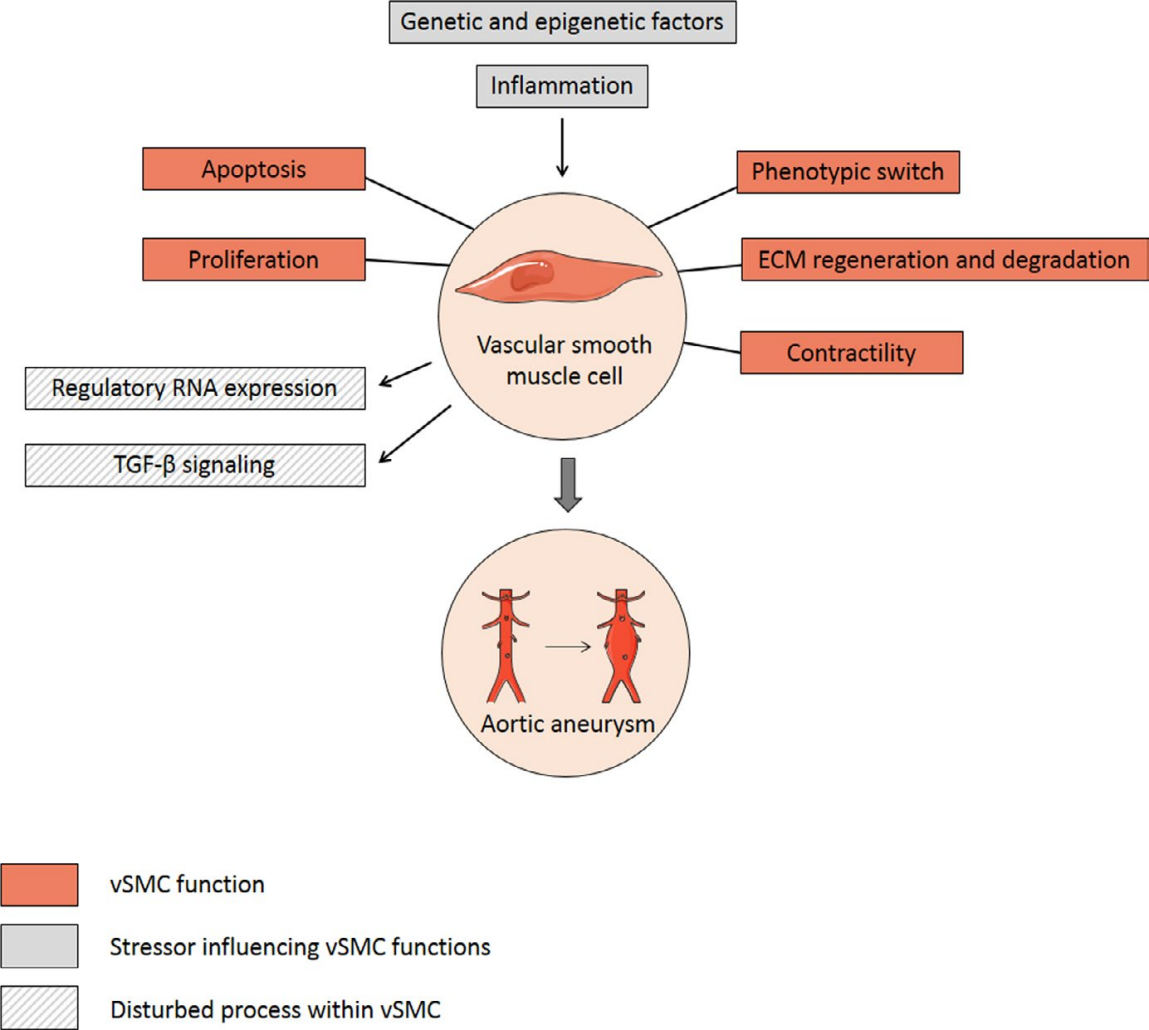
Overview of vSMC functions, stressors influencing vSMC functions and disturbed processes within vSMC during AA and AD development and progression. Apoptosis, phenotypic switch, ECM regeneration and degradation, proliferation and contractility are important functions of vSMC in the aortic wall. Dysregulation of these vSMC functions by infiltrative inflammatory cells and (epi)genetic factors can contribute to AA and AD formation. These pathological conditions activate adaptive responses within vSMC, such as changes in TGF‐β signalling and regulatory RNA expression. Abbreviations: AA, aortic aneurysm; AD, aortic dissection; ECM, extracellular matrix; RNA, ribonucleic acid; TGF‐β, transforming growth factor β; vSMC, vascular smooth muscle cell. Elements were modified from Servier Medical Art, licensed under a Creative Common Attribution 3.0 Generic License. https://smart.servier.com/; https://creativecommons.org/licenses/by/3.0/

Imbalance in vSMC phenotypic switching can contribute to several pathological processes causing cardiovascular diseases, including AA and AD.^10^ However, the exact role of vSMC dysregulation in AA and AD development and progression is complex and poorly understood. In this review, we summarize current knowledge on vSMC functions in the aortic wall and describe the potential role of vSMC dysfunction in abdominal and thoracic AA and AD development and progression. Knowledge on vSMC‐mediated pathomechanisms underlying early and advanced stages of aortic wall weakening will aid in the design of nonsurgical treatments for prevention and stabilization of AA and AD. Figure 1 gives an overview of the described vSMC functions, stressors and disturbed processes involved in progressive aortic wall weakening. A systematic database search was performed to select articles for this review and the screening process is depicted in Figure S1.

### 1.1 vSMC functions

#### 1.1.1 Apoptosis of vSMC

**FIGURE 2:**
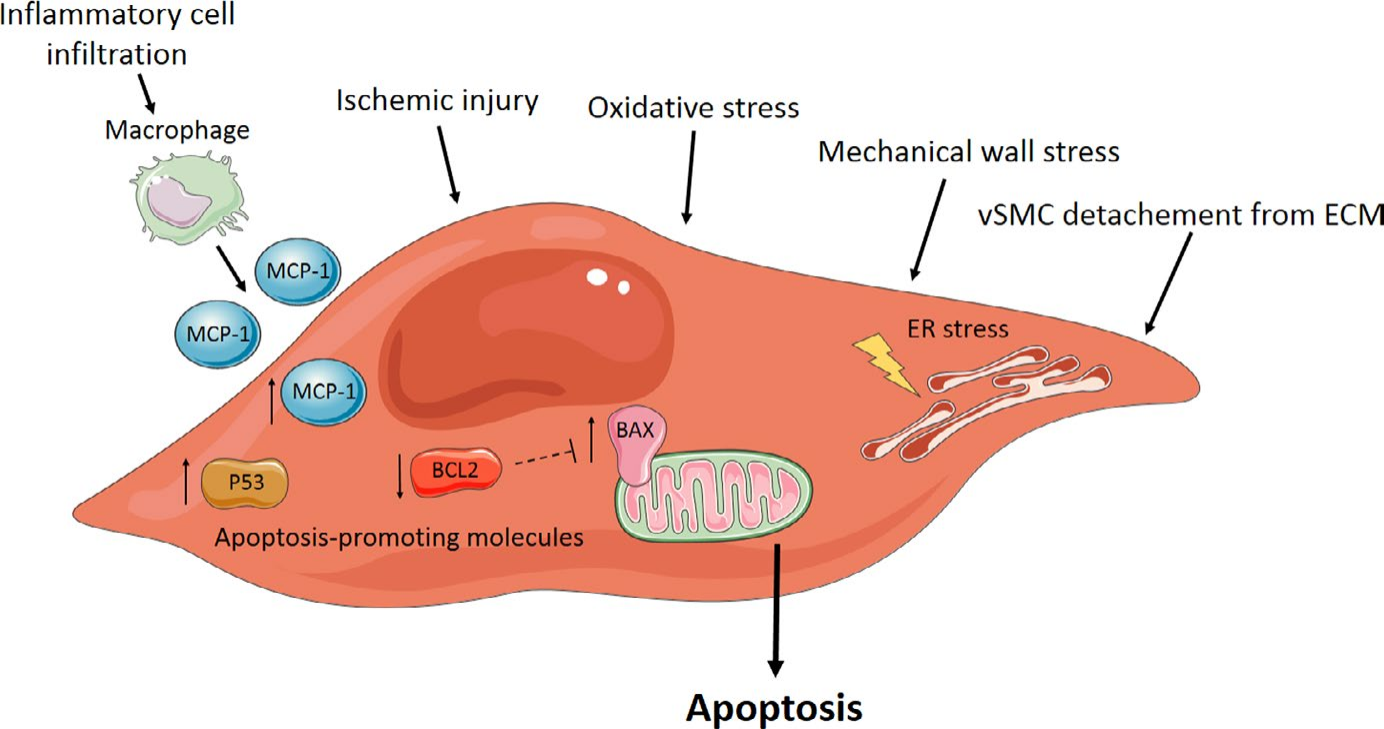
Different mechanisms to induce vSMC apoptosis within the aortic wall. Inflammatory cell infiltration into the aortic wall, ischaemic injury, oxidative stress, mechanical wall stress and detachment of the ECM can induce vSMC apoptosis. ER stress and high expression levels of MCP‐1, P53 and BAX proteins within vSMC promote vSMC apoptosis. BCL2, an inhibitor of BAX, is downregulated. Abbreviations and symbols: BAX, Bcl‐2‐associated X protein; BCL2, B‐cell lymphoma 2; ECM, extracellular matrix; ER, endoplasmic reticulum; MCP‐1, monocyte chemoattractant protein‐1; vSMC, vascular smooth muscle cell; ‐ ‐|, inhibitory effect; ↓, Decreased expression; ↑, Increased expression. Elements were modified from Servier Medical Art, licensed under a Creative Common Attribution 3.0 Generic License. https://smart.servier.com/; https://creativecommons.org/licenses/by/3.0/

The loss of vSMC in the medial layer of the aortic wall due to apoptosis is an early hallmark of AA development. Decreased vSMC density weakens the aortic wall and limits matrix repair capacity, since vSMCs are essential for ECM regeneration. The decrease in vSMC density and coincident reduction in ECM regeneration make the aortic wall more prone to dilatation and thereby contribute to development and progression of AA and AD. A decrease in vSMC number in AAA compared with healthy human aortic tissue was first demonstrated by López‐Candales et al.^11^ High levels of p53 (cell cycle arrest and apoptosis marker) observed in AAA tissue suggested that this decreased vSMC density was caused by vSMC apoptosis.^11^ Increased vSMC apoptosis in AAA tissue was described in multiple human^12^, ^13^, ^14^, ^15^ and rodent studies.^16^ Various mechanisms to induce the switch of vSMC to a more senescent phenotype with high susceptibility to apoptosis were reported and are shown in Figure 2. Inflammatory cell infiltration into the human aneurysmal aortic wall contributes to vSMC programmed cell death by production of apoptosis‐promoting molecules, such as cytokines and perforin.^13^, ^17^ An important cytokine involved in the inflammatory response in the aneurysmal aortic wall is monocyte chemoattractant protein‐1 (MCP‐1), and elevated MCP‐1 levels increased vSMC apoptosis in different AAA mouse models.^17^, ^18^, ^19^, ^20^ Oxidative stress, caused by products such as nitric oxide and oxygen‐free radicals produced by atherosclerotic plaques on the inside of the aortic wall and by vSMC, induces vSMC apoptosis in rats.^21^ Also, endoplasmic reticulum (ER) stress is demonstrated in human AAA tissue and accompanied by severe vSMC apoptosis.^22^

In the human thoracic aorta, the infiltration of inflammatory cells and ER stress induced by mechanical wall stress are associated with vSMC apoptosis, contributing to TAA and thoracic aortic dissection (TAD) development and progression.^23^, ^24^ vSMC isolated from human TAA tissue exposed to oxidative stress demonstrate pro‐apoptotic traits, such as DNA fragmentation, cellular shrinkage, membrane blebbing and chromatin condensation, indicating that oxidative stress contributes to vSMC apoptosis activation in the thoracic aorta.^25^, ^26^ vSMC apoptosis in human TAA and TAD tissue is regulated by an imbalance between pro‐apoptotic Bcl‐2‐associated X protein (BAX) and anti‐apoptotic B‐cell lymphoma 2 (BCL2) levels.^27^ Mechanical stress induced downregulation of transcriptional regulator yes‐associated protein‐1 (YAP‐1) in human thoracic aortic aneurysm and dissection (TAAD) tissue and was associated with increased vSMC apoptosis.^28^ In contrast, upregulation of YAP‐1 in a rat model has a protective effect against TAD formation by decreasing vSMC apoptosis.^29^ Heat‐shock protein (HSP)70^30^ and HSP27,^31^ which are increased in response to cellular oxidative stress in human TAA and TAD tissue respectively, also both have an anti‐apoptotic and protective effect in the thoracic aortic wall.

Apoptosis can be inhibited by transcription factor EB (TFEB), a master regulator of autophagy. However, TFEB is found to be downregulated in both human and mouse AA lesions, thereby increasing vSMC apoptosis and promoting AAA formation in different mouse models.^32^ This critical role for autophagy in regulating vSMC death is also regulated by the autophagy protein 5 gene (*ATG5*). Deletion of this gene causes loss of autophagy in vSMC and increases susceptibility of vSMC death and enhancement of ER stress‐dependent inflammation within the aortic wall.^33^

#### 1.1.2 Phenotypic switch of vSMC

**FIGURE 3:**
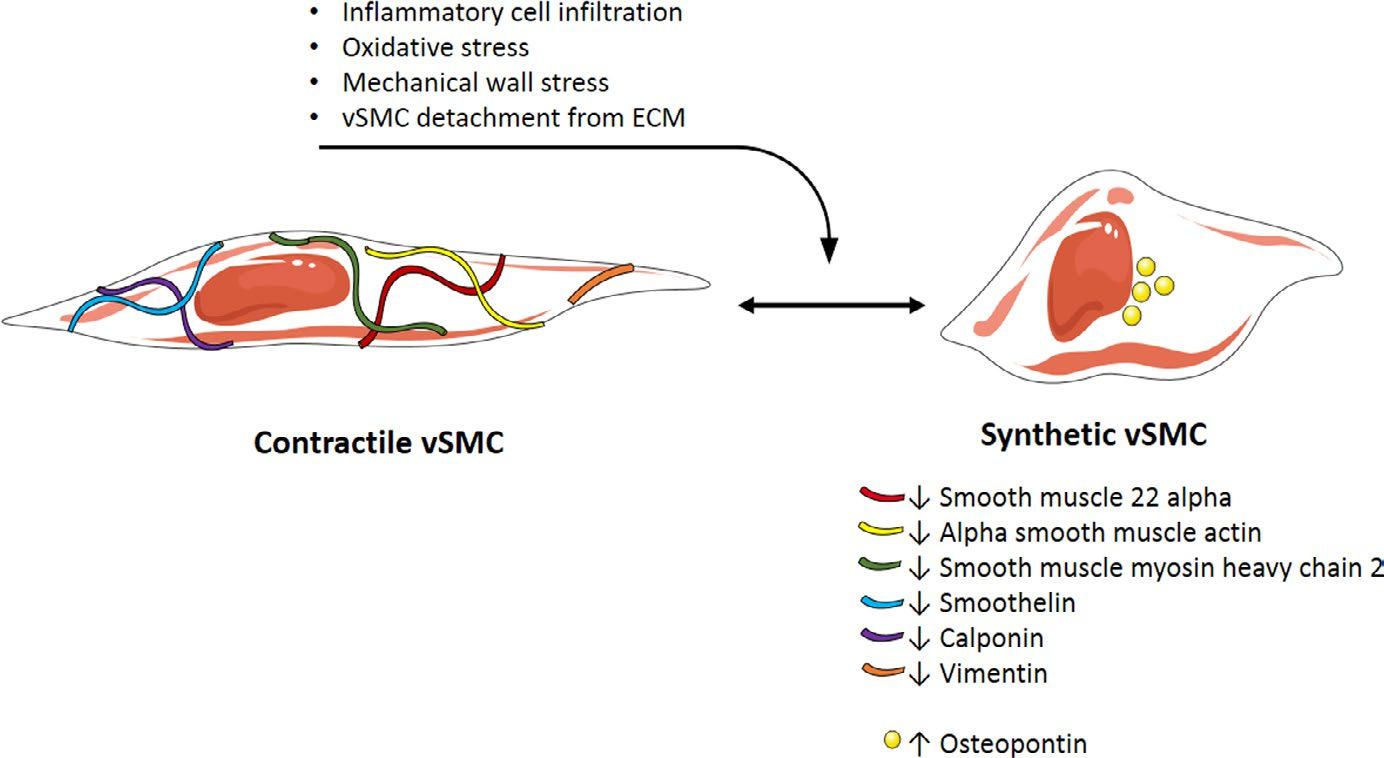
Vascular smooth muscle cell phenotypic switching. vSMC phenotypic switching can be induced by inflammatory cell infiltration, oxidative stress, mechanical wall stress or vSMC detachment from the ECM caused by ECM degradation. The switch from contractile to synthetic vSMC phenotype results in decreased expression of smooth muscle 22 alpha, alpha smooth muscle actin, smooth muscle myosin heavy chain 2, smoothelin, calponin and vimentin are decreased, while osteopontin is upregulated. Abbreviations and symbols: ECM, extracellular matrix; vSMC, vascular smooth muscle cell; ↓, Decreased expression; ↑, Increased expression. Elements were modified from Servier Medical Art, licensed under a Creative Common Attribution 3.0 Generic License. https://smart.servier.com/; https://creativecommons.org/licenses/by/3.0/

Vascular smooth muscle cells have the ability to switch from a contractile to a synthetic phenotype in the healthy aortic wall. Synthetic vSMCs are proliferative, while contractile vSMCs are more differentiated and express a higher number of SMC specific contractile markers. During AA and AD progression, the balance between contractile and synthetic vSMC is shifted towards synthetic vSMC and increased proteolytic enzyme production by those vSMC. These proteolytic enzymes can degrade the ECM, which facilitates the detachment of vSMC from the ECM and promotes vSMC migration and apoptosis. vSMC phenotypic switching is reported in AAA,^9^, ^34^, ^35^, ^36^, ^37^, ^38^, ^39^, ^40^, ^41^, ^42^, ^43^, ^44^, ^45^, ^46^, ^47^, ^48^, ^49^, ^50^ TAA^51^, ^52^, ^53^, ^54^, ^55^, ^56^, ^57^, ^58^, ^59^, ^60^, ^61^ and TAAD^62^, ^63^, ^64^, ^65^, ^66^, ^67^, ^68^, ^69^ human and rodent studies. An overview of the most important changes and inducers of vSMC phenotypic switching is indicated in Figure 3. The switch is characterized by a reduction in vSMC‐specific contractile proteins, such as smooth muscle 22alpha (SM22alpha) and alpha smooth muscle actin (αSMA) in both thoracic and abdominal aneurysmal aortic tissues.^9^, ^39^, ^51^, ^53^

vSMC phenotypic switching in the abdominal aorta was found to be initiated by inflammatory cell infiltration regulated by MCP‐1 in vitro and in a mouse model.^40^ Upregulation of osteoprotegerin (member of the cytokine receptor of tumour necrosis factor (TNF) receptor superfamily) in human AAA biopsies promoted an inflammatory vSMC phenotype with impaired proliferation and increased apoptosis.^47^, ^48^ Oxidative stress is another driver for the switch from contractile to synthetic phenotype.^39^, ^41^ Furthermore, the Notch1 pathway is known to be involved in AAA formation and vSMC‐specific haploinsufficiency of Notch1 maintains the contractile vSMC phenotype and prevents matrix remodelling in the murine abdominal aorta.^43^

In the thoracic aneurysmal aorta, smooth muscle myosin heavy chain 2 (SM‐MHC‐2), smoothelin, calponin and vimentin were also found to be reduced.^51^, ^53^, ^64^ In contrast, the expression of osteopontin, a marker for synthetic vSMC, is increased in human ascending TAA and TAD tissue.^61^, ^64^, ^65^ HSP90 is upregulated in response to cellular stress in human TAD tissue and inhibition of HSP90 in a mouse model has a protective effect on TAD formation by suppressing vSMC phenotypic switching.^69^ This is in contrast to the protective effect of upregulated HSP27 and HSP70 against vSMC apoptosis, as described above.^30^, ^31^ Hypertension causes vSMC phenotypic switching mediated by reactive oxygen species (ROS) accumulation in the human thoracic aorta, accompanied by increased mechanical wall stress.^54^ ECM‐vSMC connection is crucial for maintenance of vSMC phenotype; vSMC‐specific deletion of the ECM fibulin‐4 gene in a mouse model leads to loss of the vSMC contractile phenotype, hyperproliferation and ascending AA formation.^58^, ^59^

#### 1.1.3 ECM regeneration and degradation regulated by vSMC

**FIGURE 4:**
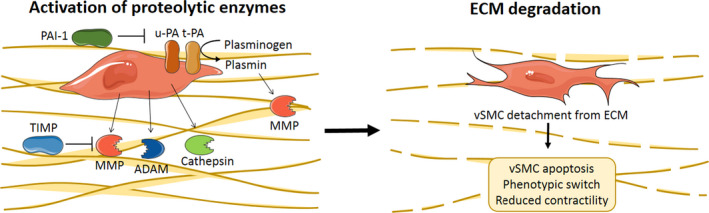
Extracellular matrix degradation by proteolytic enzymes regulated by vSMC. MMP, ADAM and cathepsins, activated and secreted by vSMC, can break down the ECM. Plasminogen can be activated into plasmin by u‐PA or t‐PA, both located on the vSMC membrane. PAI‐1 can inhibit u‐PA, and t‐PA. MMP can be inhibited by TIMP and activated by plasmin. ECM degradation causes vSMC detachment from the ECM and results in vSMC apoptosis, phenotypic switching and reduced contractility. Abbreviations: ADAM, the A disintegrin and metalloproteinase; ECM, extracellular matrix; MMP, matrix metalloprotease; PAI‐1, plasminogen activator inhibitor; TIMP, tissue inhibitors of matrix metalloproteinases; t‐PA, tissue‐type plasminogen activator; u‐PA, urokinase plasminogen activator; vSMC, vascular smooth muscle cell. Elements were modified from Servier Medical Art, licensed under a Creative Common Attribution 3.0 Generic License. https://smart.servier.com/; https://creativecommons.org/licenses/by/3.0/

Vascular smooth muscle cells are involved in regulation of the aortic wall elasticity and production of ECM proteins. Together with elastic ECM fibres, such as collagen and elastin, vSMCs form a functional unit vital for maintaining structural and functional integrity. This connection between vSMC and the ECM is essential for regulation of vSMC functions, such as vSMC contractility. However, ECM degradation occurs in aneurysmal tissue, contributing to rupture and dilation of the aortic wall. vSMCs are actively participating in this process through activation and modulation of matrix metalloproteinases (MMP), the A disintegrin and metalloproteinase (ADAM) family, cathepsins and the fibrinolytic pathway. An overview of the role of vSMC in ECM degradation is shown in Figure 4.

Matrix metalloproteinases (MMP) are endopeptidases capable of degrading ECM proteins, resulting in aortic tissue remodelling. Synthesis levels of MMP by vSMC in AAA tissue are found to be increased compared with control aortic tissues in human and rodent studies, especially of MMP‐2^70^, ^71^, ^72^, ^73^, ^74^, ^75^, ^76^, ^77^, ^78^ and MMP‐9.^70^, ^72^, ^76^, ^78^, ^79^, ^80^, ^81^ Also, other MMP, namely MMP‐1,^80^, ^82^, ^83^ MMP‐3,^83^ MMP‐7,^84^ MMP‐12^80^ and MMP‐13,^85^ are expressed by vSMC in AAA tissue. An important MMP regulator found in human AAA tissue is the activator of MMP‐2, called membrane type 1 MMP (MT‐1 MMP).^75^, ^80^, ^86^ In TAA and TAD tissue, increased levels of MMP‐1, MMP‐2 and MMP‐9 expression by vSMC compared with control are reported.^62^, ^87^, ^88^, ^89^, ^90^, ^91^ MMP‐2 has a dual role in ECM turnover in the mouse thoracic aorta: it mediates ECM degradation, as well as ECM synthesis.^92^ MMP‐17 loss of function in a genetic mouse model resulted in dysfunctional vSMC, altered ECM and increased TAA susceptibility, which suggested that proteolytic MMP‐17 activity is an important regulator of vSMC function.^93^ The secretion and activation of MMP is regulated by tissue inhibitors of matrix metalloproteinases (TIMP) expressed in AAA tissue^73^, ^77^, ^80^, ^86^, ^94^, ^95^, ^96^ and TAA and TAD tissue.^62^, ^97^

The proteolytic function of the ADAM family of metalloproteases regulated by vSMC also plays a role in ECM degradation. Similar expression levels of ADAMs 8, 9, 10, 12, 15 and 17 were found in vSMC of human AAA tissue and control aorta.^94^ In contrast, ADAM17 was upregulated in mouse AAA^98^ and human TAA tissue,^99^ and ADAM17 deficiency in vSMC had a protective function in two different mouse models.^98^, ^99^ In human TAAD tissue, increased expression of A disintegrin and metalloproteinase with thrombospondin motifs (ADAMTS)‐1 and ADAMTS‐4 localized in vSMC was reported compared with control aortic tissue. Increased levels of ADAMTS‐1 and ADAMTS‐4 were associated with more degradation of the ECM proteoglycan versican.^100^

The activation of another type of proteases, called cathepsins, can modulate the ECM and vSMC function in the aortic aneurysmal wall as well. Cathepsins B, D, G, L and S were found, localized in vSMC, in higher concentrations in human AAA tissue, compared with control.^101^, ^102^, ^103^, ^104^ Cathepsins can promote vSMC apoptosis,^105^, ^106^ elastin degradation,^105^ protease activity^106^, ^107^ and inflammatory cell accumulation.^107^ Reduced levels of the cysteine protease inhibitor cystatin C were demonstrated in human AAA lesions^108^ and complete cystatin C knockout in an AAA mouse model resulted in inflammatory cell accumulation, severe elastin fragmentation and increased vSMC apoptosis.^109^

Extracellular matrix breakdown by proteolytic enzymes and vSMC apoptosis increases arterial wall permeability and thereby the advection of plasma proteins interacting with vSMC and ECM in the aortic wall. As a result, high concentrations of plasminogen are present and activate the fibrinolytic pathway in the aortic wall. In the human abdominal aorta, the expression of tissue‐type plasminogen activator (t‐PA) on vSMC membranes can activate plasminogen into plasmin.^110^ Once active, plasmin is involved in MMP activation and fibronectin degradation, both leading to vSMC detachment from the ECM. Overexpression of plasminogen activator inhibitor (PAI‐1), an inhibitor of t‐PA and urokinase plasminogen activator (u‐PA), in an AAA rat model prevented aneurysm formation by the inhibition of plasminogen activators and MMP.^111^ In human TAA tissue, accumulation of plasminogen, t‐PA, u‐PA and plasmin was also seen, associated with vSMC.^112^ Inhibition and clearance of plasmin in human TAA tissue is regulated by plasmin‐protease nexin 1 (PN‐1) complexes internalized via low‐density lipoprotein receptor‐related protein‐1 (LRP‐1) in vSMC.^113^ Overexpression of PN‐1 and PAI‐1 mRNA and proteins in human vSMC cultures isolated from TAA tissue compared with TAD and control tissue was associated with plasmin inhibition and protection of vSMC from detachment and death.^114^

#### 1.1.4 vSMC proliferation

Vascular smooth muscle cells proliferation increases the number of vSMC within the aortic wall and can therefore have a protective effect and delay AA and AD development. However, diminished proliferative capacity is seen in human AAA‐derived vSMC compared with nonaneurysmal vSMC.^115^ Inhibition of vSMC proliferation in human AAA biopsies is caused by an G1 cell cycle arrest, regulated by urocortin‐2.^116^ High aortic flow causes aortic mechanical stretch, and this stimulates endothelial cell and vSMC proliferation in an experimental rat model.^117^, ^118^ In an AAA rabbit model, interleukin‐10 (IL‐10) treatment inhibited the inflammatory response in the aortic wall and promoted vSMC proliferation.^119^ In the tunica media of human thoracic AD patients, SM22 is downregulated, which was associated with promotion of vSMC proliferation in an in vitro assay.^120^ Furthermore, upregulated HSP27 has a protective effect on vSMC by promoting vSMC proliferation, suppressing apoptosis and protecting against elevated oxidative stress in human TAD samples.^31^

#### 1.1.5 Contractility of vSMC

The ability of vSMC within the aortic wall to contract is important to maintain vSMC/ECM tensegrity and plays a key role in mechanotransduction. Impaired contractility of vSMC of human AAA patients upon ionomycin stimulation was shown compared with control vSMC.^121^ Fibulin‐5‐deficient mice showed diminished vSMC contractility of the thoracic aorta in response to potassium loading, suggesting that sufficient vSMC‐ECM connection is essential for vSMC contraction.^122^ Contraction assays in LRP‐1‐deficient mice demonstrated attenuated vasoreactivity, which identified LRP‐1 as a critical modulator of vSMC contraction by regulating calcium signalling in the thoracic aorta.^123^

### 1.2 Stressors and disturbed processes influencing vSMC functions

#### 1.2.1 Inflammation

**FIGURE 5:**
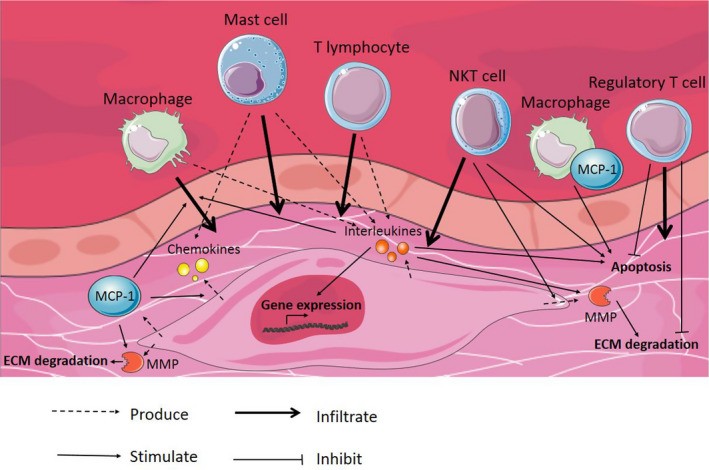
Inflammatory response in the aortic wall regulated by vSMC and affecting vSMC functions. During AA and AD progression, inflammatory cells infiltrate into the aortic wall. These inflammatory cells, together with vSMC, produce chemokines and interleukins, which subsequently activate various processes in the tunica media. vSMC start producing MMP, in turn causing ECM degradation. vSMC apoptosis is induced and gene expression in vSMC is modulated. In contrast, infiltration of regulatory T cells has a protective effect by inhibiting vSMC apoptosis and ECM degradation. Abbreviations: AA, aortic aneurysm; AD, aortic dissection; ECM, extracellular matrix; MCP‐1, monocyte chemoattractant protein‐1; MMP, matrix metalloprotease; NKT cell, natural killer T cell; vSMC, vascular smooth muscle cell. Elements were modified from Servier Medical Art, licensed under a Creative Common Attribution 3.0 Generic License. https://smart.servier.com/; https://creativecommons.org/licenses/by/3.0/

Vascular smooth muscle cells have the ability to attract inflammatory cells and induce a pro‐inflammatory state in the aortic wall. The infiltration of these inflammatory cells into the aortic wall and their release of cytokines and other mediators affect vSMC functions. An overview of the inflammatory response in the aortic wall regulated by vSMC and affecting vSMC functions during AA and AD development is depicted in Figure 5.

MCP‐1 is an important regulator of the inflammatory response during AAA development and is found in increased levels in human^124^ and mouse AAA tissue^125^, ^126^ compared with control tissue. MCP‐1 in the tunica media can increase MMP‐9 secretion by vSMC^124^ and induce more chemokine production by vSMC^125^ or macrophage accumulation within the aortic wall.^126^ An in vitro vSMC/macrophage co‐culture was used to demonstrate that macrophages primed with MCP‐1 cause higher levels of vSMC apoptosis compared with control macrophages, suggesting that MCP‐1‐primed macrophages are more cytotoxic.^127^ Lysyl oxidase (LOX) can suppress secretion of MCP‐1 in cultured vSMC, and enhanced LOX activity in an AAA mouse model prevented macrophage infiltration and AAA progression.^128^

The expression of interleukins (IL), such as IL‐5,^129^ IL‐6^126^, ^130^ and IL‐1beta (IL‐1b),^131^ in the aortic wall modulates vSMC functions and is involved in the inflammatory response contributing to AAA development. IL‐5 stimulation of mouse aortic vSMC increased MMP‐2 and MMP‐9, while IL‐5 stimulation of macrophages did not alter MMP expression.^129^ Elevated wall tension caused by hypertension resulted in IL‐6 production by vSMC, subsequently causing macrophage infiltration in an AAA mouse model.^126^ IL‐1b expression mediates an increased matrix turnover by affecting collagenase and collagen gene expression in human vSMC.^131^ Similar findings were reported for TAA tissue, where IL‐1b protein was increased approximately 20‐fold compared with control tissue. Genetic deletion of IL‐1b and IL‐1 receptor in an experimental TAA mouse model demonstrated preserved elastin and vSMC associated with fewer inflammatory cells.^132^ In TAD patients, increased IL‐6 downregulated the expression of vSMC contractile proteins alpha‐SMA and SM22alpha and induced autophagy.^133^ In human TAD samples, IL‐18 expression was increased, mainly derived from macrophages and partly from T lymphocytes and vSMC. This upregulated IL‐18 expression participated in macrophage‐induced vSMC apoptosis.^134^

The infiltration of different inflammatory cell types into the abdominal aortic wall affects vSMC functions. Accumulated mast cells in AAA mouse lesions release pro‐inflammatory cytokines IL‐6 and IFN‐gamma, which may induce vSMC apoptosis, proteolytic enzyme expression and aortic wall remodelling.^135^ In vitro studies with human vSMC showed that both T and natural killer T (NKT) cells adhere to vSMC. While CD4+ T cells enhance vSMC proliferation, CD4+ CD161+ NKT cells inhibit vSMC proliferation by inducing apoptosis,^136^ which suggest that these cells contribute to AAA development. In contrast, regulatory T cells were shown to have a protective effect in an AAA mouse model. Regulatory T cells decreased pro‐inflammatory cytokines levels, MMP‐2 and MMP‐9 expression, vSMC apoptosis and oxidative stress, while the expression of the anti‐inflammatory IL‐10 and TGF‐β was increased.^137^ Furthermore, regulatory T‐cell treatment in an AAA mouse model decreased expression of the pro‐inflammatory protein cyclooxygenase‐2 in vSMC and macrophages and increased vSMC viability.^138^ Patients suffering from bicuspid aortic valve (BAV) and tricuspid aortic valve (TAV) disease have an increased risk for TAAD development and rupture of the aortic wall. When comparing gene expression profiles of human BAV and TAV tissue biopsy samples, an immune response activation is only seen in the aortic media of TAV patients, suggesting that inflammation is involved in TAA formation in TAV patients, but not in BAV patients.^139^

#### 1.2.2 Genetic and epigenetic factors

**FIGURE 6:**
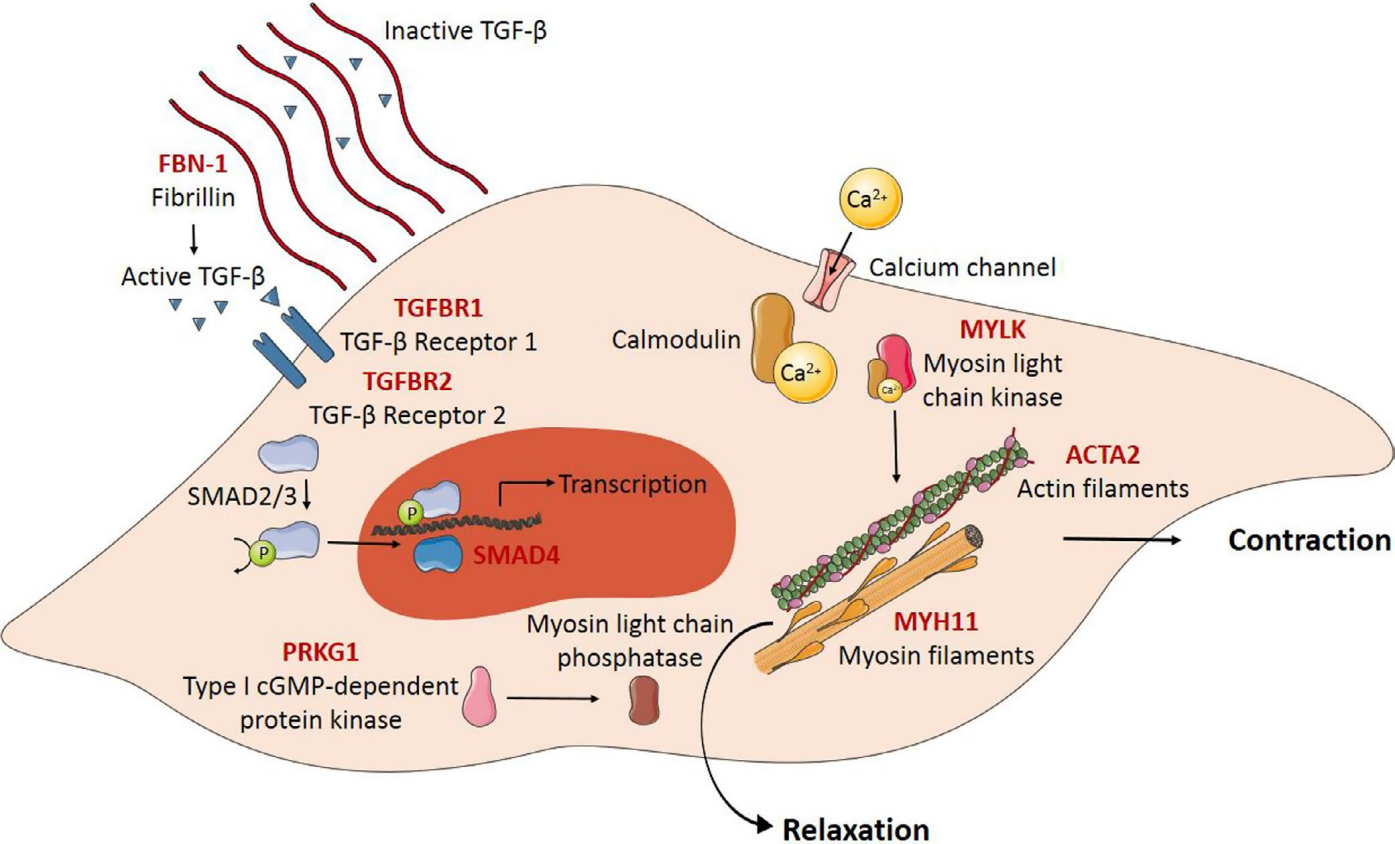
Overview of the contractile apparatus in vSMC and mutations affecting vSMC functions causing TAAD formation. The contraction of vSMC is initiated by Ca^2+^ influx, binding to calmodulin. This Ca^2+^/Calmodulin complex binds to myosin light chain kinase, which phosphorylates the myosin filaments and results in contraction. Decrease in the Ca^2+^ concentration within in the cell inactivates myosin light chain kinase and de‐phosphorylation of the myosin filaments, controlled by type I cGMP‐dependent protein kinase, results in vSMC relaxation. Mutations in genes encoding for vSMC contractile apparatus proteins (indicated in red) result in reduced force generation. Mutations in genes encoding for the large glycoprotein fibrillin‐1 (indicated in red) affect the structure of microfibrils and result in increased active TGF‐β levels. Mutations in the TGF‐β signalling pathway and downstream proteins (indicated in red) can also contribute to TAAD formation by affecting transcription. Abbreviations: Ca^2+^, calcium ion; P, phosphate; TAAD, thoracic aortic aneurysm and dissection; TGFBR, transforming growth factor β receptor; TGF‐β, transforming growth factor β; vSMC, vascular smooth muscle cell. Elements were modified from Servier Medical Art, licensed under a Creative Common Attribution 3.0 Generic License. https://smart.servier.com/; https://creativecommons.org/licenses/by/3.0/

Thoracic aortic aneurysm and dissection pathogenesis is often driven by a genetic component, affecting proteins encoding for the vSMC contractile apparatus and the ECM in the tunica media. In contrast to TAAD, little is known about genetic causes of AAA.^140^ A highly efficient method to investigate the pathogenicity of variants of vSMC contractile apparatus proteins is the use of SMC‐like cells generated by transdifferentiation of human dermal fibroblasts.^141^ Figure 6 and Table 1 show an overview of mutations affecting vSMC functions and causing TAAD formation.

**TABLE 1:** Overview of known genetic causes of familiar TAAD and the affected processes within the aortic wall

Gene	Affected process within the aortic wall	Reference
ACTA2	vSMC contractility	142, 143, 144, 145, 146, 147, 148, 149, 150, 151
MYH11	vSMC contractility	148, 152, 153, 154, 155
PRKG1	vSMC contractility	156, 157
MYLK	vSMC contractility	158
FBN1 (Marfan syndrome)	ECM organization vSMC phenotypic switching	159 162
TGFB1/2 (Loeys‐Dietz syndrome)	vSMC contractility vSMC phenotypic switching	174 180
SMAD4	vSMC apoptosis vSMC contractility ECM regeneration and degradation	177, 178
COL3A1 (Ehlers‐Danlos syndrome type IV)	ECM organization	170
LOX	ECM organization	171
MFAP5	ECM organization	172
FOXE3	vSMC apoptosis and survival	183

Abbreviations: ECM, extracellular matrix; TAAD, thoracic aortic aneurysm and dissection; vSMC, vascular smooth muscle cell.

##### Mutations affecting the vSMC contractile apparatus

The vSMC contractile apparatus consists of thin SMC‐specific filaments α‐actin (SM α‐actin, encoded by *ACTA2*) and thick SMC‐specific filament myosin heavy chain dimer (MHC, encoded by *MYH11*), assembled with two regulatory light chains (LC) and two essential LC. vSMC contraction is initiated by calcium (Ca^2+^) influx, binding to calmodulin. This Ca^2+^/calmodulin complex binds to myosin LC kinase (MLCK, encoded by *MYLK*), leading to phosphorylation of the regulatory LC of the myosin filaments. The force‐generating cycle is activated by myosin ATPase, and the myosin motor heads move along the actin filaments. After vSMC contraction, a decrease in Ca^2+^ concentration within the cell inactivates MLCK. The myosin regulatory LC is de‐phosphorylated by myosin light LC phosphatase (MLCP), which is controlled by type I cGMP‐dependent protein kinase (PKG‐1, encoded by *PRKG1*). A schematic overview of the vSMC contraction is shown in Figure 6.

Mutations in genes encoding the vSMC contractile proteins result in reduced force generation and destabilizing the contractile apparatus, which contributes to the development of TAADs. The most common mutations causing familial TAAD are found in *ACTA2*, causing 14% of all cases.^142^, ^143^, ^144^, ^145^, ^146^, ^147^, ^148^, ^149^, ^150^, ^151^ Loss of SM α‐actin filaments in vSMC causes decreased integrin recruitment for cell‐matrix adhesion. This reduced interaction between vSMC and the ECM combined with reduced contractility makes these vSMCs unable to generate force.^144^ Mutations in *MYH11*, encoding the other main protein of the vSMC contractile apparatus myosin, alter contractile vSMC function and may contribute to TAAD development.^148^, ^152^, ^153^, ^154^, ^155^ Heterozygous gain‐of‐function mutations in *PRKG1* are also associated with TAAD.^156^, ^157^ Increased PKG‐1 activity resulted in decreased phosphorylation of myosin regulatory LC and thereby decreased vSMC contraction.^156^ Lastly, heterozygous loss‐of‐function mutations in *MYLK* are associated with familial TAAD.^158^

##### Mutations affecting the ECM

The contractile apparatus in vSMC is anchored to the ECM by integrin‐containing focal adhesions, called dense plaques. Integrin receptors in these dense plaques bind to elastin through microfibrils, with the large glycoprotein fibrillin‐1 (encoded by *FBN1*) as the main protein in microfibrils. Heterozygous *FBN1* mutations cause the connective tissue disorder Marfan syndrome. Marfan patients tend to be tall, slender and have with hyper‐flexible joints, accompanied by serious complications in organs containing connective tissue. Marfan disease also affects the connective tissue in the aortic wall, which makes them susceptible for TAA, but not AAA formation, at younger age compared with nongenetic TAA patients.

The aortic wall in Marfan patients contains low levels of fibrillin‐1 and undifferentiated vSMC.^159^ vSMC detachment from the ECM causes vSMC phenotypic switching and increases expression of MMP‐9 and elastin in a Marfan mouse model.^160^ vSMC cultured from Marfan mice showed a more mesenchymal‐like phenotype with impaired force‐generating capacity due to impaired cytoskeleton and focal adhesion organization.^161^ These phenotypic changes are also seen in human Marfan vSMC, affecting SM α‐actin, smoothelin, SM22 alpha, calponin‐1 and myocardin (transcription factor essential for vSMC‐specific differentiation), and resulting in greater cellular and ECM stiffness.^162^ These studies suggested that loss of vSMC attachment caused by the *FBN1* mutations activates a nonproductive programme to synthesize and remodel the ECM.

Fibrillin‐1 does not only play a role in the structural integrity of the aortic wall, but also regulates the bioavailability of TGF‐β. Fibrillin‐1 and microfibrils can together create a reservoir for TGF‐β, maintaining TGF‐β there in an inactive from. Mutations in fibrillin‐1 disrupt the biological scaffolding role of fibrillin‐1, causing an increase in active TGF‐β, thereby activating TGF‐β receptor‐mediated Smad signalling.^163^ Increased TGF‐β expression is shown in vSMC of Marfan patients compared with healthy subjects,^164^, ^165^ leading to the activation of canonical SMAD3 signalling^164^ and the noncanonical ERK signalling.^166^ The activation of these pathways can model vSMC proliferation, apoptosis, inflammation and phenotypic switching. Furthermore, increased TGF‐β in Marfan patients induced vSMC senescence through excessive ROS generation^165^ and increased vSMC apoptosis.^167^ The potential role of TGF‐β signalling in AA and AD formation is further described below.

Reactive oxygen species production in vSMC of Marfan mice was found to be anatomically specific, with increased NADPH activity in vSMC derived from the ascending aorta compared with vSMC derived from the descending aorta.^168^ ROS produced by vSMC in the tunica media of Marfan patients targets actin‐based cytoskeleton components and regulators of ECM homeostasis.^169^

There are more known mutations affecting ECM proteins, subsequently disturbing vSMC functions and initiating AA or AD development. Ehlers‐Danlos syndrome type IV is characterized by an *COL3A1* mutation.^170^ A mutation in the *LOX* gene, encoding for an enzyme that normally cross‐links collagen and elastin molecules in the aortic wall, causes a loss of stability and elasticity of the aortic wall.^171^ Loss‐of‐function mutations in *MFAP5*, encoding for microfibril‐associated glycoprotein 2, which is a component of the ECM interacting with fibrillin‐1, also appear as an underlying cause of familiar TAAD development.^172^

##### Mutations affecting TGF‐β signalling

Loeys‐Dietz syndrome is characterized by mutations in genes encoding for components of the TFG‐β pathway, including TGF‐β receptor (TGFBR)‐1 and 2.^173^ The role of the altered TGF‐β signalling pathway on vSMC function during AA and AD development and progression is complex, and controversial findings have been reported. vSMC explanted from patients with heterozygous mutations in TGFBR2 (encoded by *TGFBR2*) showed decreased expression of vSMC contractile proteins, which may contribute to TAAD by affecting the contractile function of vSMC.^174^ vSMC‐specific deletion of *TGFBR2* in adult mice resulted in mild thoracic aneurysmal degeneration, while vSMC‐specific deletion of *TGFBR1* in the same mouse model caused severe thoracic aneurysmal degeneration.^175^ In contrast to these findings, vSMC‐specific *TGFBR2* disruption in an AAA mouse model prevented AAA formation by reduced elastin degradation, vSMC loss, macrophage infiltration and MMP expression.^176^ An explanation for these contrasting effects of TGF‐β on the aortic wall can be the different embryological origins of abdominal and thoracic vSMC. Also, genetic variants of SMAD4, the intracellular secondary messenger of the TGF‐β pathway, are reported as promotors of vSMC apoptosis, proteoglycan degradation and reduced contractile protein gene expression in human TAAD pathogenesis.^177^, ^178^ Likewise, SMAD3‐deficient mice developed TAA rapidly by the activation of immune responses, but did not show vSMC loss or MMP activation in vSMC.^179^

In human TAD tissue, TGF‐β stimulation was found to contribute to aortic wall weakening by iinducing vSMC phenotypic switching from a contractile to a synthetic phenotype.^180^ In human AAA tissue, TGF‐β mRNA and protein expression are found to be increased compared with control tissue.^181^, ^182^ This overexpression of TGF‐β was associated with reduced vSMC density, caused by more vSMC apoptosis, while vSMC proliferation was reduced.^182^ In contrast, another study reports no changes in proliferation of aneurysmal rat vSMC treated with TGF‐β.^78^

##### Mutations affecting other vSMC functions

*FOXE3* encodes for a transcription factor regulating anti‐apoptotic and pro‐survival pathways and *FOXE3* mutations were found to contribute to TAAD development.^183^ *FOXE3*‐deficient mice showed reduced vSMC density and impaired vSMC differentiation in the ascending aorta.^183^

##### Epigenetic changes in vSMC

Not only genetic changes but also epigenetic changes in vSMC are related to AAA,^184^, ^185^, ^186^ TAA^187^, ^188^ and TAAD^189^ pathogenesis. In contrast to genetics, epigenetic changes can modify gene expression without altering the genetic code itself, for example by DNA methylation or histone modification. Sirtuin‐1 is a class III histone deacetylase which is decreased in human AAA samples.^186^ Knockout of Sirtuin‐1 in vSMC accelerated formation and rupture of AAA by inducing inflammation and vascular senescence, while Sirtuin‐1 overexpression in vSMC had a protective effect in a mouse model.^186^ vSMC collected from human TAA tissue showed increased SMAD2 expression compared with vSMC collected from healthy thoracic tissue, which was dependent on epigenetic regulation of the SMAD2 promotors involving histone modifications.^187^, ^188^ Enhancer of zeste homolog 2 (EZH2) is a methyl transferase of histone H3 that functions as a transcriptional repressor and inhibits autophagic cell death of vSMC during TAAD progression.^189^ Epigenetic changes in AA pathogenesis are of interest since these changes can be reversible and altered by environmental factors, such as smoking. Epigenetic changes may therefore explain the link between AA risk factors and AA development.

#### 1.2.3 Regulatory RNAs regulating vSMC functions

*MicroRNA* (miRNA) are RNA strands of 19–24 nucleotides regulating gene expression, which are involved in AA and AD pathogenesis by modulating different vSMC functions. Table 2 gives an overview of miRNA involved in regulating vSMC functions, and how expression level of these miRNA affects vSMC function. The anatomical location and the species in which these miRNAs were studied are also shown. In AAA pathogenesis, miRNA‐21,^190^ miRNA‐26a,^191^ miRNA‐28‐5p,^192^ miRNA‐129‐5p,^193^ miRNA‐155‐5p,^194^ miRNA‐195^195^ and miRNA‐504^196^ are reported to be involved in regulation of vSMC apoptosis. miRNA‐155,^197^ miRNA‐195,^198^ miRNA‐205^199^ and miRNA‐516a‐5p^200^ are found to be involved in ECM regeneration and degradation. miRNA‐21,^190^ miRNA‐129‐5p,^193^ miRNA‐155,^197^ miRNA‐195^195^ and miRNA‐504^196^ can regulate vSMC proliferation, while miRNA‐155^197^ can regulate vSMC migration. miRNA‐24,^201^ miRNA‐33,^202^ miRNA‐155^197^ and miRNA‐195^198^ are involved in regulation of the inflammatory response within the aortic wall.

**TABLE 2:** Overview of miRNA and lnc‐RNA involved in regulation of vSMC functions during AAA, TAA, TAAD and TAD development

vSMC function	Regulated by	Expression level and effect miRNA/lnc‐RNA	Location	Species	Reference
vSMC apoptosis	miRNA‐21 miRNA‐26a miRNA‐28‐5p miRNA‐129‐5p miRNA‐155‐5p miRNA‐195	Increased during AAA development, miRNA overexpression decreased apoptosis Decreased during AAA development, miRNA upregulation decreased apoptosis Increased during AAA development, miRNA acts as an apoptosis driver Decreased during AAA development, miRNA overexpression enhanced apoptosis Expression during AAA development not mentioned, inhibiting miRNA induced apoptosis Increased during AAA development, miRNA enhanced apoptosis	AAA AAA AAA AAA AAA AAA	Humans and mouse Humans Humans Humans and mouse Humans Humans	190 191 192 193 194 195
	miRNA‐504 miRNA‐26b	Decreased during AAA development, miRNA overexpression decreased apoptosis Decreased during TAD development, miRNA overexpression decreased apoptosis	AAA TAD	Humans Humans	196 204
	miRNA‐145	Decreased during TAD development, miRNA downregulation enhanced apoptosis,^205^ while miRNA overexpression decreased apoptosis^206^	TAD	Humans Humans and rat	205 206
	miRNA‐320d	Decreased during TAD development, miRNA overexpression enhanced apoptosis	TAD	Humans	207
	miRNA‐582	Decreased during TAD development, miRNA overexpression enhanced apoptosis	TAD	Humans	207
	lnc‐RNA NEAT1	Increased during AAA development, lnc‐RNA knockdown decreased apoptosis, while lnc‐RNA overexpression enhanced apoptosis	AAA	Not mentioned	216
	lnc‐RNA GAS5	Increased during AAA development, lnc‐RNA overexpression enhanced apoptosis	AAA	Humans and mouse	217
	lnc‐RNA H19	Increased during AAA development, lnc‐RNA knockdown decreased apoptosis, while overexpression had the opposite effect	AAA	Mouse	218
	lnc‐RNA PVT1	Increased during AAA development, lnc‐RNA knockdown decreased apoptosis	AAA	Humans and mouse	219
	lnc‐RNA LUCAT1	Increased during AAA development, lnc‐RNA depletion decreased apoptosis, while lnc‐RNA promotion enhanced apoptosis	AAA	Not mentioned	220
	lnc‐RNA LINC00473	Increased during AAA development, lnc‐RNA overexpression enhanced apoptosis	AAA	Humans	221
	lnc‐RNA LOXL1‐AS	Increased during TAA development, lnc‐RNA overexpression decreased apoptosis	TAA	Humans	222
	lnc‐RNA MIAT	Increased during TAA development, lnc‐RNA overexpression decreased apoptosis	TAA	Humans	223
	lnc‐RNA HOTAIR	Decreased during TAA development, lnc‐RNA knockdown enhanced apoptosis	TAA	Humans	224
	lnc‐RNA HIF 1alpha antisense RNA	Expression during TAA development not mentioned, lnc‐RNA suppression decreased apoptosis	TAA	Humans	225
	linc‐RNA p‐21	Increased during TAA development, linc‐RNA overexpression enhanced apoptosis	TAA	Humans	226
vSMC phenotypic switch	miRNA‐124 miRNA‐134‐5p miRNA‐143/145 cluster miRNA‐21	Decreased during TAD development, miRNA overexpression enhanced phenotypic switch (decrease in contractile genes) Decreased during TAD development, miRNA overexpression increased contractile phenotype Decreased during TAD development, miRNA knockdown induced phenotypic switch Increased during TAAD development, miRNA knockdown increased switch from contractile to synthetic phenotype, due to dysfunctional TGF‐β signalling	TAD TAD TAD TAAD	Humans Humans and mouse Humans Mouse	208 209 210 215
ECM regeneration and degradation	miRNA‐155 miRNA‐195 miRNA‐205 miRNA‐516a‐5p miRNA‐145 miRNA‐30a lnc‐RNA PVT1 lnc‐RNA HOTAIR	Increased during AAA development, miRNA overexpression enhanced MMP‐2, MPP‐9 protein expression, while inhibition had the opposite effect Increased during AAA development, miRNA overexpression enhanced MMP‐2 and MMP‐9 protein expression Increased during AAA development, miRNA overexpression reduced LRP‐1 protein expression, and subsequent reduced MMP‐9 protein clearance Expression during AAA development not mentioned, miRNA overexpression increased MMP‐2 protein and decreased TIMP‐1 protein expression, while knockdown had the opposite effect Increased during TAA development, miRNA overexpression enhanced OPN and collagen III protein, while inhibition had the opposite effect Increased during TAD development, miRNA overexpression decreased LOX and elastin protein expression Increased during AAA development, lnc‐RNA knockdown suppressed ECM disruption Decreased during TAA development, lnc‐RNA knockdown decreased collagen types I and III mRNA and protein expression	AAA AAA AAA AAA TAA TAD AAA TAA	Humans and mouse Humans Humans Humans Humans Humans and rat Humans and mouse Humans	197 198 199 200 203 214 219 224
vSMC proliferation	miRNA‐21 miRNA‐129‐5p miRNA‐155 miRNA‐195 miRNA‐504 miRNA‐26b miRNA‐124 miRNA‐133	Increased during AAA development, miRNA overexpression enhanced proliferation Decreased during AAA development, miRNA overexpression decreased proliferation Increased during AAA development, miRNA overexpression enhanced proliferation, while inhibition had the opposite effect Decreased during AAA development, miRNA inhibited proliferation Decreased during TAD development, miRNA overexpression promoted proliferation Decreased during TAD development, miRNA overexpression enhanced proliferation, while knockdown inhibited proliferation Decreased during TAD development, miRNA overexpression enhanced proliferation Decreased during TAD development, miRNA upregulation decreased proliferation	AAA AAA AAA AAA AAA TAD TAD TAD	Humans and mouse Humans and mouse Humans and mouse Humans Humans Humans Humans Humans	190 193 197 195 196 204 208 212
	miRNA‐145 miRNA‐146a‐5p	Decreased during TAD development, miRNA downregulation enhanced proliferation,^205^ and miRNA overexpression promoted proliferation^206^ Increased during TAD development, miRNA overexpression enhanced proliferation	TAD TAD	Humans Humans and rat Humans	205 206 213
	lnc‐RNA NEAT1	Increased during AAA development, lnc‐RNA knockdown enhanced proliferation, lnc‐RNA overexpression decreased proliferation	AAA	Not mentioned	216
	lnc‐RNA GAS5 lnc‐RNA LUCAT1	Increased during AAA development, lnc‐RNA overexpression decreased proliferation Increased during AAA development, lnc‐RNA depletion enhanced proliferation, while lnc‐RNA promotion decreased proliferation	AAA AAA	Humans and mouse Not mentioned	217 220
	lnc‐RNA LINC00473	Increased during AAA development, lnc‐RNA overexpression decreased proliferation	AAA	Humans	221
	lnc‐RNA LOXL1‐AS lnc‐RNA HOTAIR	Increased during TAA development, lnc‐RNA overexpression enhanced proliferation Decreased during TAA development, lnc‐RNA knockdown decreased proliferation	TAA TAA	Humans Humans	222 224
	lnc‐RNA HIF 1alpha antisense RNA	Expression during TAA development not mentioned, lnc‐RNA suppression enhanced proliferation	TAA	Humans	225
	linc‐RNA p‐21	Increased during TAA development, linc‐RNA overexpression decreased proliferation	TAA	Humans	226
vSMC migration	miRNA‐155	Increased during AAA development, miRNA overexpression enhanced migration, while inhibition had the opposite effect	AAA	Humans and mouse	197
	miRNA‐27a miRNA‐133 miRNA‐134‐5p	Decreased during TAD development, miRNA downregulation decreased migration Decreased during TAD development, miRNA upregulation decreased migration Decreased during TAD development, miRNA overexpression decreased migration	TAD TAD TAD	Humans and mouse Humans Humans and mouse	211 212 209
	miRNA‐145 miRNA‐146a‐5p	Decreased during TAD development, miRNA downregulation enhanced migration Increased during TAD development, miRNA overexpression enhanced migration	TAD TAD	Humans Humans	205 213
Inflammatory response within the aortic wall	miRNA‐24	Decreased during AAA development, miRNA downregulation enhanced pro‐inflammatory response	AAA	Humans and mouse	201
	miRNA‐33 miRNA‐155 miRNA‐195 lnc‐RNA PVT1	Increased during AAA development, miRNA knockdown reduced MCP‐1 mRNA and protein expression Increased during AAA development, miRNA overexpression enhanced MCP‐1 protein expression, while inhibition had the opposite effect Increased during AAA development, miRNA overexpression enhanced IL‐1β and IL‐6 expression Increased during AAA development, lnc‐RNA knockdown decreased pro‐inflammatory cytokines	AAA AAA AAA AAA	Humans and mouse Humans and mouse Humans Humans and mouse	202 197 198 219

Abbreviations: AAA, abdominal aortic aneurysm; ECM, extracellular matrix; linc‐RNA, long intergenic noncoding RNA; lnc‐RNA, long noncoding RNA; miRNA, microRNA; RNA, ribonucleic acid; TAA, thoracic aortic aneurysm; TAAD, thoracic aortic aneurysm and dissection; TAD, thoracic aortic dissection; vSMC, vascular smooth muscle cell.

In TAA pathogenesis, only miRNA‐145^203^ is reported as regulator of ECM remodelling. In TAD pathogenesis, miRNA‐26b,^204^ miRNA‐145,^205^, ^206^ miRNA‐320d and miRNA‐582^207^ can regulate vSMC apoptosis. miRNA‐124,^208^ miRNA‐134‐5p^209^ and miRNA‐143/145 gene cluster^210^ can have a role in regulation of vSMC phenotypic switching. miRNA‐27a,^211^ miRNA‐133,^212^ miRNA‐134‐5p,^209^ miRNA‐145^205^ and miRNA‐146a‐5p^213^ are found to be involved in vSMC migration. miRNA‐26b,^204^ miRNA‐124,^208^ miRNA‐133,^212^ miRNA‐145^205^, ^206^ and miRNA‐146a‐5p^213^ are reported as regulators of vSMC proliferation. miRNA‐30a is found to be involved in ECM remodelling by vSMC.^214^ In a TAAD mouse model, knockout of miRNA‐21 induced a switch from a contractile to a synthetic vSMC phenotype, due to dysfunctional TGF‐β signalling.^215^

Long noncoding RNA (lnc‐RNA) is defined as transcripts exceeding 200 nucleotides which are not translated into protein. However, they are crucial participants in both AAA and TAA pathogenesis by altering vSMC functions. Table 2 shows an overview of lnc‐RNA involved in regulating vSMC functions during AAA and TAA development and how expression level of these lnc‐RNA affect vSMC function. The anatomical location and the species in which these lnc‐RNA were studied are also shown. In AAA pathogenesis, vSMC apoptosis can be regulated by lnc‐RNA NEAT1,^216^ GAS5,^217^ H19,^218^ plasmacytoma variant translocation 1 (PVT1),^219^ LUCAT1^220^ and LINC00473.^221^ Lnc‐RNA PVT1 is also a regulator of ECM regeneration and degradation, and the inflammatory response within the aortic wall.^219^ Regulators of vSMC proliferation are lnc‐RNA NEAT1,^216^ GAS5,^217^ LUCAT1^220^ and LINC00473.^221^ In TAA pathogenesis, lnc‐RNA LOXL1‐AS,^222^ myocardial infarction associated transcript (MIAT),^223^ HOX transcript antisense intergenic RNA (HOTAIR),^224^ HIF 1alpha antisense RNA^225^ and long intergenic noncoding RNA (linc‐RNA) p‐21^226^ are associated with regulation of vSMC apoptosis. Lnc‐RNA LOXL1‐AS,^222^ HOTAIR,^224^ HIF 1alpha antisense RNA^225^ and linc‐RNA p‐21^226^ are reported be involved in regulation of vSMC proliferation, and lnc‐RNA HOTAIR is involved in ECM remodelling.^224^

## 2 DISCUSSION AND FUTURE PERSPECTIVES

AA are pathological dilations of the aortic wall, which can rupture and cause internal bleeding. As summarized in this review, previous studies have shown that vSMC dysfunction has a paramount role in aortic wall weakening and subsequent AA and AD formation. Although several attempts have been made to develop pharmacological treatment options for stabilization or prevention of AA and AD, none of them has led to a broadly applicable treatment yet. In case of type B AD, patients are treated with anti‐hypertensive medication but the majority of the patients do not show improvement upon this therapy.^7^ Disease progression is unpredictable, which makes it challenging to decide whether patients need surgical repair to prevent rupture. Therefore, more research into the underlying molecular mechanism of vSMC dysfunction in AA and AD progression and development is warranted.

### 2.1 Treatment options based on rodent studies

Targeting the loss of vSMC within the aortic wall caused by apoptosis is a potential treatment strategy. During endovascular repair, a graft is placed within the aortic wall, and seeding these endovascular grafts with vSMC has been shown to have a protective and reparative effect in a rat model. vSMC graft seeding prevented AAA formation, by decreasing elastin degradation and monocyte infiltration in AAA‐prone rats.^227^ Likewise, aneurysm diameter stabilized after endovascular seeding of vSMC with aneurysmal aortic xenografts in an AAA rat model by inhibition of the ECM degradation process. The expression of MMP‐1, MMP‐3, MMP‐7, MMP‐9 and MMP‐12 decreased, while the expression of TIMP‐1, TIMP‐2 and TIMP‐3 increased after vSMC seeding.^228^ The importance of vSMC in the aortic wall is highlighted by these studies and demonstrates a protective, paracrine effect of endovascular vSMC seeding on the aortic wall. Endovascular AA stent repair combined with the restoration of the healing capability of vSMC could be a promising therapeutic alternative.

The production and activation of proteolytic enzymes by vSMC contribute to AA progression and could therefore be a potential treatment target. Inhibition of ECM degradation by Doxycycline, a nonspecific inhibitor of MMP, gave promising results in rat and mouse studies.^229^, ^230^ However, evidence from clinical studies to confirm this beneficial effect is still lacking and this MMP inhibitor is therefore not used as therapeutic treatment for AA patients yet.^231^, ^232^ Upregulation of other inhibitors of proteolytic enzymes, such as PAI‐1 and TIMP, using gene therapy may also decrease ECM degradation within the aortic wall. Local overexpression of TIMP‐1 in a rat aorta resulted in decreased MMP‐9 and activated MMP‐2, which preserved elastin in the tunica media and prevented aneurysmal degeneration and rupture.^233^ Another treatment option would be to target external factors which affect vSMC functions, such as inflammation or oxidative stress. Statins, which are lipid‐lowering drugs, have previously been shown to suppress AAA growth in a mouse model, by having an anti‐inflammatory effect on the aortic wall and reducing ER stress and apoptosis.^234^ In humans, an association between statin use and a decreased expansion rate of infrarenal AA was also reported, but this still needs to be confirmed by randomized clinical trials.^235^ Additionally, the ability of regulatory miRNA and lnc‐RNA expression to alter gene expression and modulate vSMC functions may be explored to improve vSMC function within the aortic wall and prevent AA formation. miRNA activity can be manipulated by anti‐miRs, blockmiRs or miRNA mimics, delivered by viral vectors, nanoparticles or liposomes. This potential of miRNA as a treatment option was previously shown to be effective for other cardiovascular diseases.^236^

### 2.2 Strength of tissue biobanks, patient‐derived vSMC and in vitro aortic models

So far, none of the above‐described proposed therapeutic treatment options are implemented in the clinic. AA is a heterogeneous and multifactorial disorder with differences in origin and disease progression in every patient, which makes the development of therapeutic AA treatments challenging. To address this challenge, it is necessary to perform in vitro experiments and translational studies on a large number of patient samples. Creating a biobank with patient samples, and specifically vSMC, is needed to define the link between defects in vSMC and clinical characteristics in large cohorts of patients.^237^ Our group showed in vitro that contraction of vSMC isolated from AAA patients is impaired compared with healthy control vSMC.^121^ Impaired vSMC contraction was mainly seen in patients who were current smokers and who underwent open repair surgery after earlier endovascular repair.^121^ However, the underlying mechanism of vSMC contractility reduction leading to AA formation still remains unexplored. More research is needed into the exact proteins and pathways contributing to vSMC contractile dysfunction and the link between these molecular defects and other patient characteristics, such as sex, age and diabetes. Functional studies in human vSMC should be combined with multiple –omics studies (RNA‐seq, proteomics) in large numbers of patient compared with control samples to identify proteins and pathways involved in AA progression, which can be used as potential treatment targets.

Another explanation for the absence of therapeutic AA treatments is the lack of adequate in vitro models. These models, mimicking human disease, can be used to investigate disease progression and effects of therapeutic interventions. vSMC can be generated from stem cells, but this is a time‐consuming and expensive process. Segments of isolated aortic tissue from large animals, maintained in organ culture, can also be used as an in vitro model. This model has the advantage that laminar shear stress can be applied, but the disadvantages that these isolated aortic segments can only be kept in culture for a limited time period.^238^ Preclinical 3D cell culture models seeded with patient‐specific SMC can recreate the complex micro‐environment of the aorta in vitro. This model gives the option to study mechanical properties, fibre orientation, ECM production by vSMC and the interaction with other cell types in the aortic wall, such as endothelial cells.^239^ However, in this model, the cells lose their original tubular form. Therefore, the complexity of this model can be increased by generation of tubular grafts using a bioreactor to study additional parameters, such as flow.^240^ Another method to study aneurysmal disease in vitro is by transdifferentiation of fibroblasts, isolated from skin tissue, into vSMC‐like cells. After conversion of these dermal fibroblasts, mRNA expression of SMC markers within the vSMC‐like cells is comparable to primary human aortic SMC.^141^ This noninvasive method is highly efficient to detect gene mutations and molecular defects in vSMC‐like cells, already at an early disease stage before the patient develops AA. Specific molecular defects within individual patients can be diagnosed, and targeting these patient‐specific defects can improve patient outcome.^241^ Furthermore, functional measurements of vSMC‐like cells could have the potential to eventually predict characteristics of AA progression, such as aneurysm size growth and chance of rupture, in patients with early AA who have not yet undergone surgery. The above‐mentioned in vitro models, using either biological materials or biological combined with synthetic materials, have an important role in ‘proof of concept studies’, but they require further validation to optimize in vitro aneurysmal disease research.

A last important point to underscore is that recent studies suggest that also endothelial cells play a key role in development and progression of AA and AD. Endothelial dysfunction can activate aortic wall remodelling by release of proteases, or induction of inflammatory and oxidative stress responses.^242^, ^243^, ^244^ Therefore, we want to highlight the importance to consider the interaction between endothelial cells and vSMC while studying aneurysmal disease.

## 3 CONCLUSION

In conclusion, our review illustrates the central role of impaired vSMC function in AA and AD development and progression and highlights the stressors and molecular pathways which may be targets for therapeutic interventions. Future research should focus on studies in models, which mimic the individual AA patient and define effects of stressors and interventions in these specific patients. This way, new potential targets for pharmaceutical treatments can be found and better disease progression prediction can be made for both AA and AD in order to improve patient outcome.

## CONFLICT OF INTEREST

Not applicable.

## AUTHORS’ CONTRIBUTIONS

KR designed and performed the database searches, selected articles found in the database search, wrote the manuscript and designed the figures. TM selected articles found in the database search and revised the manuscript. JCFK designed and performed the database searches. NB, JvV and KKY helped, advised and assisted on the selection of the articles, writing of the manuscript and revised the manuscript. All authors read and approved the final manuscript.

## Supporting information

Fig S1
