# Supplementary figures and images for: The role of vascular smooth muscle cells in the development of aortic aneurysms and dissections
Source: Eur J Clin Invest. 2021 Nov 21;52(4):e13697. doi: 10.1111/eci.13697 (PMC9285394; doi:10.1111/eci.13697)

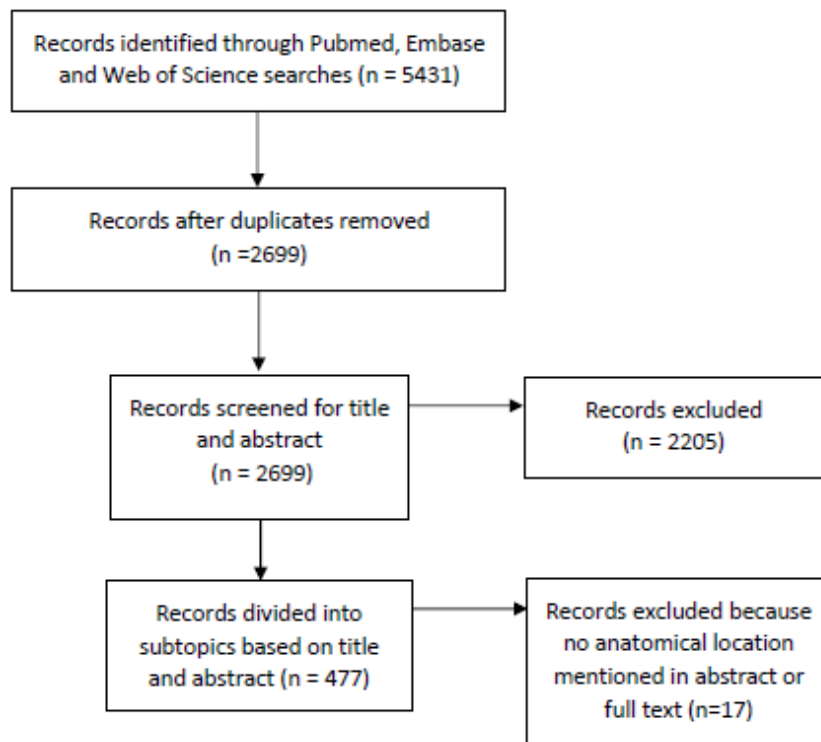

***Supplementary Figure 1: Overview of the screening process***

Supplement: Supplementary file 1 — Fig S1 [file ECI-52-0-s001.pdf]
